# Association of Cigarette Use and Substance Use Disorders among US Adults with and without a Recent Diagnosis of Cancer

**DOI:** 10.3390/curroncol28010011

**Published:** 2020-12-12

**Authors:** Joanna M. Streck, Maria A. Parker, Andrea H. Weinberger, Nancy A. Rigotti, Elyse R. Park

**Affiliations:** 1 Tobacco Research and Treatment Center, Division of General Internal Medicine, Department of Medicine, Harvard Medical School, Massachusetts General Hospital, Boston, MA 02114, USA; nrigotti@partners.org (N.A.R.); epark@mgh.harvard.edu (E.R.P.); 2 Department of Psychiatry, Harvard Medical School, Massachusetts General Hospital, Boston, MA 02114, USA; 3 Department of Epidemiology & Biostatistics, School of Public Health-Bloomington, Indiana University, Bloomington, IN 47405, USA; map2@iu.edu; 4 Ferkauf Graduate School of Psychology, Yeshiva University, Bronx, NY 10461, USA; andrea.weinberger@yu.edu; 5 Department of Epidemiology & Population Health, Albert Einstein College of Medicine, Bronx, NY 10461, USA

**Keywords:** cancer, substance use disorders, comorbidity, epidemiology, cigarette smoking

## Abstract

Background: Few studies have examined substance use disorders (SUDs) in cancer patients and it is unclear whether SUDs differentially impact cigarette smoking in patients with vs. without cancer. This study used epidemiological data to estimate current cigarette smoking prevalence and quit ratios among US adults with and without SUDs by cancer status. Methods: Data were drawn from the 2015–2018 National Survey on Drug Use and Health (*n* = 170,111). Weighted current smoking prevalence and quit ratios were estimated across survey years by SUDs (with vs. without) and by cancer status (with vs. without). Results: Among those with cancer, current smoking prevalence was higher for those with vs. without SUDs (47% vs. 13%, *p* < 0.001) and quit ratios lower for those with vs. without SUDs (45% vs. 71%, *p* = 0.002). A similar pattern was observed in adults without cancer, with higher smoking prevalence (56% vs. 21%, *p* < 0.001) and lower quit ratios (23% vs. 51%, *p* < 0.001) observed for those with vs. without SUDs, respectively. In adjusted logistic regressions, the SUD × cancer status interaction was not significant for smoking prevalence or quit ratios (AOR = 1.2; 95% CI: 0.7, 2.1, *p* = 0.56; AOR = 1.0; 95% CI: 0.5, 2.0, *p* = 0.91, respectively), though smoking prevalence was lower and quit ratios higher for adults with vs. without cancer (*ps* < 0.05). Conclusions: Among US adults with and without cancer, individuals with SUDs evidenced higher cigarette smoking and lower quit ratios than those without SUDs. Addressing SUDs and their impact on smoking cessation is critical in cancer patients with implications for improving health and treatment outcomes.

## 1. Introduction

In the United States (US) and many other countries worldwide (e.g., Canada, China), cigarette smoking is the leading cause of preventable death [1,2]. Whereas in the US general population, the prevalence of cigarette smoking has declined in recent years (~14% in 2018) [3] and quit rates have increased, many individuals continue to smoke cigarettes despite the significant health risks [1,2] associated with smoking.

Individuals with cancer constitute one group particularly vulnerable to the negative health consequences of smoking [4,5,6]. Population-based smoking prevalence estimates among cancer patients vary widely, with some studies reporting a lower smoking prevalence in cancer vs. noncancer patients, [7,8] while other studies have reported a higher smoking prevalence, particularly for those with a smoking-related vs. non-smoking-related cancer [9,10]. The most recent (2017) estimates suggest a generally equivalent prevalence of smoking in cancer vs. noncancer patients (i.e., 13% vs. 14%) [10].

Individuals with substance use disorders (SUDs) represent another population highly vulnerable to tobacco addiction and related consequences, with a high prevalence of smoking, poorer cessation outcomes, and increased tobacco-related mortality compared to the general population [11,12,13]. Indeed, smoking prevalence has increased over time in US adults with vs. without SUDs (2002 to 2014), with a higher smoking prevalence seen among those with (56%) vs. without SUDs (18%) in 2014 [13].

Additionally, SUDs are not uncommon in cancer patients, with prevalence estimates of SUDs in this population ranging from 2% to 35%, though a thorough understanding of rates and risk factors is lacking [14]. Further, very few prior studies have examined prevalence of individual substances in cancer patients, as most studies have collapsed all substances together to examine SUDs more broadly. Substance use and comorbid cancer are associated with several adverse consequences, including complications over the course of cancer treatment and the onset of other infections and medical complications [15].

To our knowledge, no prior studies have comprehensively investigated the relationship of nontobacco SUDs to cigarette smoking prevalence or quitting by cancer status. This question is critically important to address given the substantial impact of SUDs on smoking behavior in the general population and the urgent need to better understand persistent smoking in cancer patients due to the substantial cancer-related and general risks of smoking in this group. Thus, the aim of the present investigation was to use combined epidemiological data from 2015 to 2018 to estimate cigarette smoking prevalence and quit ratios among US adults with and without SUDs and to examine whether the association between SUDs and smoking prevalence and quit ratios differed by cancer diagnosis status.

## 2. Materials and Methods

### 2.1. Study Sample

Data were drawn from the 2015–2018 National Survey on Drug Use and Health (NSDUH) public use data files. The NSDUH is a cross-sectional US-representative (50 states plus District of Columbia) dataset among noninstitutionalized persons age 12 and older. The current analyses included individuals aged 18 or above (combined n for 2015–2018 = 170,111). Details of the NSDUH methodology have been previously published [16]. Briefly, in the NSDUH, household addresses are randomly selected for participation through multistage sampling. Each year, the NSDUH conducts interviews with selected individuals within a household. Individuals are surveyed at a single timepoint and the sampling procedures are designed to minimize the likelihood of individuals to completing the survey more than once. Participants complete the interviews in their home online via a computer and/or in-person with assistance from a NSDUH interviewer. Participants receive $30 (USD) compensation for completion of the interview.

### 2.2. Measures

Past-month cigarette smoking prevalence: Current cigarette smoking was assessed at each survey year. For the analyses of smoking prevalence, participants reporting smoking at least 100 lifetime cigarettes and all or part of a cigarette in the last 30 days were considered to have current (past-month) smoking [17].

Cigarette quit ratio: Cigarette quit ratios were defined as the ratio of those with former smoking to those with ever smoking at each survey year. For the analyses of quit ratios, former smoking was defined as smoking at least 100 lifetime cigarettes and no smoking in the past year. Ever smoking was defined as smoking at least 100 lifetime cigarettes [17].

Past-year cancer diagnosis: Respondents were asked whether they had a past-year cancer diagnosis at each survey year (i.e., “Did you have cancer during the past 12 months?”) and were categorized as having or not having a past-year cancer diagnosis.

Past-year nontobacco SUDs: Past-year SUDs, defined as a diagnosis of abuse and/or dependence, were assessed according to the *Diagnostic and Statistical Manual*, 4th edition (DSM-IV) [18] criteria for a range of substances (i.e., alcohol, cannabis, methamphetamines, hallucinogens, inhalants, tranquilizers, cocaine, heroin, prescription pain relievers, simulants, and sedatives). Individuals with one or more SUDs were categorized as having a SUD and those without any past-year SUDs were categorized as not having SUDs.

Demographic characteristics: Age (18–25 years, 26–34 years, 35+ years), gender (male, female), race/ethnicity (non-Hispanic (NH) White, NH Black, Hispanic, NH Other (NH Native American/Alaskan Native, NH Hawaiian/Pacific Islander, NH Asian, NH more than one race)), and annual income (<USD 20,000, USD 20,000–49,999, USD 50,000–74,999, ≥USD 75,000) were included as covariates.

### 2.3. Statistical Analyses

Demographic and substance use characteristics were examined by past-year cancer diagnosis status (with vs. without) using weighted frequencies. We estimated the weighted prevalence of each substance as well as the number of SUDs by cancer diagnosis status. Weighted current smoking prevalence and quit ratios were estimated across survey years by SUDs (with vs. without) and by past-year cancer diagnosis status. In post-estimation exploratory data analyses, adjusted logistic regression analyses tested the SUD status × past-year cancer diagnosis status interactions on current smoking prevalence and quit ratios, adjusting for age, gender, race/ethnicity, and annual income. For all analyses, data are combined across survey years (2015–2018). All analyses were conducted in Stata 16 using the “svy” procedure, which accounts for the complex sampling design of the NSDUH, interdependence of survey observations, and analysis weights (Stata Corp, 2015, College Station, TX, USA).

## 3. Results

### 3.1. Sample Description (Combined Data 2015–2018)

Participant characteristics are presented in Table 1. A significantly higher percentage of those with vs. without cancer identified as NH White (vs. NH Black, Hispanic, or Other race) and were 35 years of age or older (vs. 18–25 or 26–34 years of age). Additionally, those with a past-year cancer had significantly lower rates of past-year SUDs compared to those without a past-year cancer. When examining specific types of SUDs by cancer status, adults with a past-year cancer had lower prevalence of each SUD compared to those without a past-year cancer and also reported using a lower number of substances compared to those without a past-year cancer (Table 1).

### 3.2. Current Cigarette Smoking Prevalence (Combined Data 2015–2018)

**Figure 1:**
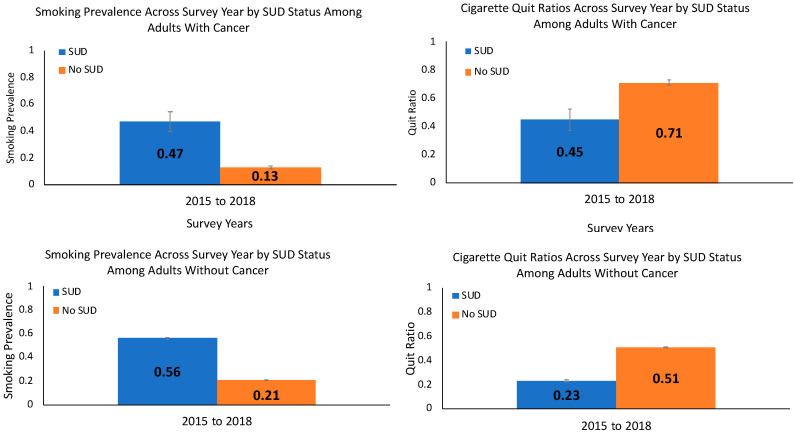
Current smoking prevalence and quit ratios (weighted %, standard error of the mean) among adults with a past-year cancer (upper panels) and without a past-year cancer (lower panels) by substance use disorder (SUD) status in 2015–2018. Notes: Data are from the 2015–2018 National Surveys on Drug Use and Health. The SUDs × cancer diagnosis status interaction was not significant for smoking prevalence (adjusted odds ratio (AOR) = 1.2; 95% confidence interval (CI): 0.7, 2.1; *p* = 0.56) or quit ratios (AOR = 1.0; 95% CI: 0.5, 2.0; *p* = 0.91) in logistic regression analyses that adjusted for age, gender, race/ethnicity, and annual income.

Current smoking prevalence was higher among those with vs. without SUDs for those with past-year cancer (SUDs: 47% vs. no SUDs: 13%, across years, *p* < 0.001; Figure 1, upper left panel) as well as for those without past-year cancer (56% vs. 21% across years, *p* < 0.001; Figure 1, lower left panel). Taken together, the smoking prevalence was lower for adults with vs. without cancer (*p* < 0.001), and for both adults with and without cancer, the smoking prevalence was higher for persons with vs. without SUDs (*p* < 0.001). In adjusted logistic regression analyses, the SUD × cancer diagnosis status interaction was not significant (AOR = 1.2; 95% CI: 0.7, 2.1; *p* = 0.56), indicating that past-year cancer diagnosis status did not modify the association of SUDs and smoking prevalence.

### 3.3. Cigarette Quit Ratios (Combined Data 2015–2018)

For those with past-year cancer, the quit ratio was lower among those with vs. without SUDs (SUDs: 45% vs. no SUDS: 71%, across years, *p* = 0.002; Figure 1, upper right panel) with a similar pattern seen for those without cancer (23% vs. 51% across years, *p* < 0.001; Figure 1, lower right panel). For both those with and without SUDs, the quit ratio was higher for adults with vs. without a past-year cancer (*p* < 0.001), and for both adults with and without cancer, the quit ratios were lower for those with vs. without SUDs (*p* < 0.001). The SUD × cancer status interaction was not significant for quit ratios (AOR = 1.0; 95% CI: 0.5, 2.0; *p* = 0.91), indicating that cancer diagnosis status did not modify the association of SUDs and cigarette quit ratios.

## 4. Discussion

This investigation used combined epidemiological data from 2015 to 2018 to estimate cigarette current smoking prevalence and quit ratios among US adults with and without SUDs by past-year cancer status. Among US adults with and without cancer, individuals with SUDs evidenced significantly higher cigarette smoking rates and lower quit ratios than those without SUDs. Overall, adults with a cancer diagnosis had lower rates of smoking and higher rates of quitting than adults without cancer, regardless of SUD status. Our estimate of smoking prevalence in patients with cancer (and without SUDs) is largely consistent with the most recent population-based estimate of adults with cancer using the National Health Interview Survey from 2017 (i.e., 13% prevalence), as well as other estimates of smoking prevalence from community-based samples (e.g., 14–15%) [10,19,20,21]. We also found a lower prevalence of SUDs in adults with versus without cancer.

Our study’s findings of lower rates of smoking and higher rates of quitting in individuals with vs. without cancer is consistent with prior research showing smoking behavior change at and following a cancer diagnosis [22]. Furthermore, our study demonstrates that the pattern seen in the general population of higher rates of smoking and lower rates of quitting among individuals with SUDs also holds for adults with cancer. The American Society of Clinical Oncology recommends screening and treating alcohol and tobacco use in cancer patients [23,24]. However, there is little consensus on how to address poly-SUDs in this population nor guidelines on the best practices for addressing SUDs and co-occurring tobacco use. Importantly, prior research in noncancer patients suggests that smoking cessation does not negatively impact substance use outcomes (e.g., abstinence) and may even improve comorbid substance use outcomes [25]. Addressing co-occurring SUDs in oncology treatment is important for improving tobacco cessation rates and subsequent health and treatment outcomes. Future research is needed to determine the optimal timing and interventions for smoking cessation in cancer patients with co-occurring SUDs.

Our study was limited in that our data were cross-sectional, and thus we could not infer causality. Additionally, cancer diagnosis was self-reported in the past year, which may have limited accuracy in reporting compared with objective confirmations of a diagnosis and/or may have not captured individuals with a history of cancer [26]. Further, we were unable to distinguish between smoking-related and non-smoking-related cancers or examine results by specific cancer type. Additionally, while our data is nationally representative of the US adult population, our findings may not be generalizable to institutionalized adults, non-US adults, or other individuals not sampled in the NSDUH. Last, the power for assessing interactions is often low in epidemiologic studies, which may be the case for the interaction between SUDs and cancer. However, the samples were large enough to estimate both the current cigarette smoking prevalence and quit ratios, which would not be possible in smaller samples. These limitations notwithstanding, to our knowledge, this is the first report to examine the relationship of SUDs to cigarette smoking prevalence and quit ratios in adults with cancer using nationally representative data. US adults with cancer demonstrated a similar pattern of results to US adults without cancer, such that individuals with SUDs had a significantly higher prevalence of cigarette smoking and lower quit ratios than those without SUDs. Smoking cessation remains an urgent priority for cancer patients given the adverse impact of smoking on cancer treatment outcomes and evidence that cessation can improve survival. A greater focus should be placed on addressing co-occurring SUDs and their impact on smoking cessation in cancer patients. Cancer treatment provides an important opportunity to screen and refer patients for SUD and smoking treatment or integrate these services into existing care.

## 5. Conclusions

Among US adults with and without a past-year self-reported cancer, individuals with SUDs evidenced higher current cigarette smoking and lower quit ratios than those without SUDs. Individuals with past-year cancer had a lower prevalence of past-year SUDs compared to those without cancer. Addressing SUDs and their impact on smoking cessation is critical in cancer patients with implications for improving health and cancer treatment outcomes including survival.

## Figures and Tables

**Table 1:** Participant characteristics by past-year cancer diagnosis status. Data from the 2015–2018 National Surveys on Drug Use and Health.

	Cancer (Unweighted n = 1571) ^a^	No Cancer (Unweighted n = 168,540) ^b^	*p* Value ^c^
**Demographics**			
Gender			0.34
Male	50.1%	48.2%	
Female	49.9%	51.8%	
Age (years)			<0.001
18–25	0.9%	14.2%	
26–34	2.8%	16.1%	
35+	96.3%	69.7%	
Race/Ethnicity			<0.001
NH White	83.7%	64.0%	
NH Black	7.1%	11.9%	
Hispanic	3.3%	8.1%	
NH Other ^d^	5.8%	16.0%	
Income (annual)			0.26
<USD 20,000	14.5%	16.6%	
USD 20,000–49,999	30.5%	29.7%	
USD 50,000–74,999	17.3%	16.0%	
≥USD 75,000	37.7%	37.8%	
**Substance Use Disorders**			
Any past-year SUD	4.6%	7.9%	<0.001
1 SUD	4.0%	6.6%	
2+ SUDs	0.4%	1.2%	
Alcohol Use Disorder	3.4%	6.0%	0.001
Cannabis Use Disorder	0.2%	1.4%	<0.001
Opioid Use Disorder	0.8%	0.8%	0.87
Stimulant Use Disorder ^e^	0.2%	0.5%	0.01
Other Use Disorder ^f^	0.4%	0.7%	0.11
Past-month cigarette smoking	15%	24%	<0.001

Tabled values represent weighted percentages unadjusted for other demographic characteristics and may not add to 100% due to rounding. NH, non-Hispanic; SUD, substance use disorder. ^a^ Weighted *n* = 3,971,316. ^b^ Weighted *n* = 239,667,703. ^c^ *p*-values compare characteristic values for persons with a past-year cancer diagnosis vs. without a past-year cancer diagnosis. ^d^ NH Other category included individuals who identified as NH Native American/Alaskan Native, NH Hawaiian/Pacific Islander, NH Asian, or NH more than one race. ^e^ Includes prescription stimulant and cocaine use disorder. ^f^ Includes hallucinogen, inhalant, methamphetamine, tranquilizer, or sedative use disorder.
